# Significance of Immune and Non-Immune Cell Stroma as a Microenvironment of Hepatocellular Carcinoma—From Inflammation to Hepatocellular Carcinoma Progression

**DOI:** 10.3390/ijms251910233

**Published:** 2024-09-24

**Authors:** Jacek Baj, Magdalena Kołodziej, Joanna Kobak, Jacek Januszewski, Kinga Syty, Piero Portincasa, Alicja Forma

**Affiliations:** 1 Department of Correct, Clinical and Imaging Anatomy, Chair of Fundamental Sciences, Medical University of Lublin, Jaczewskiego 4, 20-090 Lublin, Poland; jacek.baj@umlub.pl (J.B.); jacek.januszewski000@gmail.com (J.J.); 2 Chair and Department of Forensic Medicine, Medical University of Lublin, Jaczewskiego 8b, 20-090 Lublin, Poland; magdalenakolodziej502@gmail.com (M.K.); kobak.joannaelzbieta@gmail.com (J.K.); 3 Institute of Health Sciences, John Paul the II Catholic University of Lublin, Konstantynów 1G, 20-708 Lublin, Poland; kinga.syty@kul.pl; 4 Clinica Medica “A. Murri”, Department of Biomedical Sciences & Human Oncology, University of Bari Medical School, 70124 Bari, Italy; piero.portincasa@uniba.it

**Keywords:** hepatocellular carcinoma, HCC, immune stroma, tumor-infiltrating neutrophils, bone-marrow-derived cells, tumor-associated mast cells, cancer-associated fibroblasts, tumor-associated macrophages, liver-sinusoidal endothelial cells, lymphocytes

## Abstract

Hepatocellular carcinoma (HCC) is the most common liver cancer as well as the most prevalent cause of death in the adult patient population with cirrhosis. The occurrence of HCC is primarily caused by chronic liver inflammation that might occur because of a viral infection, non-alcoholic fatty liver disease (NAFLD), or various lifestyle-associated factors. The objective of this review was to summarize the current knowledge regarding the microenvironment of HCC, indicating how immune- and non-immune-cell stroma might affect the onset and progression of HCC. Therefore, in the following narrative review, we described the role of tumor-infiltrating neutrophils, bone-marrow-derived cells, tumor-associated mast cells, cancer-associated fibroblasts, tumor-associated macrophages, liver-sinusoidal endothelial cells, lymphocytes, and certain cytokines in liver inflammation and the further progression to HCC. A better understanding of the HCC microenvironment might be crucial to introducing novel treatment strategies or combined therapies that could lead to more effective clinical outcomes.

## 1. Introduction

Liver cancer is a big challenge for today’s medicine, being the sixth most commonly diagnosed cancer worldwide [1]. An increased occurrence of hepatocellular carcinoma (HCC) (70% of all diagnosed HCC) is reported in Asia, probably due to the sanitary conditions and a higher number of hepatitis B virus (HBV) infection incidents [2]. Although the most common type of liver cancer is metastasis of colorectal cancer, HCC represents 80% of all primary liver malignancies with a total of 747,000 cases of HCC globally in 2019 [3,4]. This aggressive cancer is the most frequent primary liver tumor which originates in hepatocytes. Due to personal predispositions and exposure to different carcinogens, the response of the immunological system can be promoted. Consequent inflammation caused by hepatic stellate cells and natural killer cells may lead to hepatic cell death and promote the occurrence of liver cirrhosis and fibrosis. The occurrence of recurring liver cell repopulation and the constant impact of toxic and carcinogenic factors might affect the proper function of hepatocytes and lead to HCC development [5,6]. Furthermore, the metabolic microenvironment also plays an important role in HCC immune evasion. Tumor cells respond to environmental stressors, such as energy competitiveness, suppressive metabolites, and hypoxia, by undergoing metabolic adaptations to survive apoptosis. Such mechanisms may potentially be significant contributors to HCC progression [7]. Early stages of HCC are usually asymptomatic and may be found in routine imaging examinations often commissioned for different reasons. Patients with more advanced stages of HCC may present symptoms such as jaundice, weight loss, malaise, fever, ascites, or even hepatic encephalopathy [8,9]. The diagnosis of HCC can be based on specific symptoms revealed in radiological examinations. Due to ongoing angiogenesis in a lesion, HCC presents more arterial vascularization than the surrounding liver tissue in a CT, which is called a ‘’wash-in effect’’. The portal supply gradually decreases, resulting in less portal blood within the lesion relative to the encompassing liver tissue during the venous phases, which is called a ‘’wash-out’’ [9]. Another factor that can be useful during HCC diagnostic processes is the alpha-fetoprotein (AFP). Although its sensitivity in detecting the early stages of HCC may be helpful in case of inconclusive radiological situations [10], a liver biopsy is the surest procedure for an HCC diagnosis. However, due to its invasiveness, it is commissioned only in concrete cases [11]. The therapy for HCC consists of different types of treatment. Liver transplantation, tumor resection, or transarterial embolization (TACE) are common types of surgical treatment methods for HCC [10,12,13]. Also, a combined therapy involving atezolizumab (anti-programmed death-ligand 1 (PD-L1)) and bevacizumab (anti-vascular endothelial growth factor (VEGF)) presents acceptable effects in HCC treatment [14].

## 2. HCC Etiology

As mentioned above, HCC is primarily related to inflammatory processes, cirrhosis, and fibrosis of the liver. Such pathological mechanisms may develop due to different risk factors and toxic substances. Alcohol consumption is one of the biggest factors in cirrhosis development and a subsequent HCC occurrence [15]. Taniai M presented that the risk of cirrhosis becomes significant with drinking 20 g/day of alcohol for 10 years in women and 60-80 g/day of alcohol for 10 years in men [16]. Viral infections might also contribute to HCC development, including chronic infections of HBV, hepatitis C virus (HCV), and hepatitis D virus (HDV) [17]. McGlynn et al. reported that an inflammatory response due to an HBV infection and the integration of viral DNA can lead to chronic necrosis, cirrhosis, and, consequently, the development of HCC [17,18]. To eradicate HBV, a robust response of CD4^+^ T and CD8^+^ T cells might be generated. HBV-specific CD8^+^ T lymphocytes have antiviral efficacy through either destroying the infected hepatocytes directly or generating TNF-α and IFN-γ. Moreover, T cells co-stimulate B cells, which then results in the production of antibodies against the HBV surface antigen (HBsAg), HBV core antigen (HBcAg), and HBV antigen (HBeAg). All these actions lead to a limitation of the HBV infection and viral expansion. However, it might not lead to a regression of the infection and, instead, result in a chronic HBV infection, during which continuous immune responses may lead to recurrent liver injuries and its reconstruction. Such mechanisms might potentially become factors contributing to HCC onset [19,20]. HCV RNA does not integrate with the human genome but can cause chronic infection which also plays a significant role in HCC development [18]. Obesity may also correlate with an increased risk of HCC onset [21]. Metabolic abnormalities associated with poor eating habits may lead to the development of diseases such as metabolic-dysfunction fatty liver disease (MAFLD) which replaced the term of non-alcoholic fatty liver disease (NAFLD) in 2020 due to its more appropriate definition. A MAFLD diagnosis is based on the same criteria as hepatic steatosis, which means ≥5% of hepatocytes have lipid vacuoles when no other significant cause is present, but it adds dysregulatory factors that may disrupt homeostasis and proper liver functions. The MAFLD criteria classify type 2 diabetes and overweight or obesity with ethnic-specific body mass index (BMI) categories as the metabolic risk drivers. In addition to type 2 diabetes and overweight/obesity, there is another dysregulatory route included in the process of MAFLD development. People with a correct body weight are obligated to present two of the seven risk factors for a diagnosis to be made. These seven risk factors include: blood pressure, waist circumference, plasma high-density lipoprotein cholesterol, plasma triglycerides, insulin resistance score, prediabetes, and plasma high sensitivity C-reactive protein [21,22]. The term NAFLD, defined as the inherence of hepatic steatosis in more than 5% of hepatocytes with the absence of significant alcohol consumption and without other liver disease, is still frequently used in the literature in relation to HCC [23,24]. Another important factor of HCC onset is toxins, especially aflatoxin B1 produced by Aspergillus fungi. Consumption of aflatoxin B1 impacts genetic mutations and may accelerate the process of carcinogenesis. The promutagenic effect of aflatoxin B1 on human DNA leads to its destabilization and mutation in the p53 suppressor gene. A P53 suppressor gene mutation is known as a common genetic abnormality in HCC development [25,26]. Immunosuppression might contribute to HCC development. The coinfection of HIV and HBV without proper therapy might advance the cirrhosis of the liver and lead to a faster HCC development [27,28].

## 3. Hepatocellular Carcinoma Microenvironment

### 3.1. Tumor-Infiltrating Neutrophils (TINs)

Currently accepted theories indicate that neutrophils exhibit plasticity in cancer, thereby depending on various factors in the tumor microenvironment or the blood and bone marrow, they may develop pro-tumor or anti-tumor properties [29,30]. The significance of tumor-infiltrating neutrophils (TINs) in various cancer types, inter alia, biliary, esophageal, gastric, breast, or renal cell carcinoma has been explored, which revealed a wide range of mechanisms affecting tumor metastasis, disease progression, prediction therapeutic outcome, or prognosis [31,32,33,34,35,36]; however, TINs validity in the hepatocellular carcinoma appears to not be sufficiently investigated yet [37]. Lin et al. (2021), in their systematic review and meta-analysis, demonstrated that a high baseline neutrophil-to-lymphocyte ratio (NLR) leads to a poor prognosis or a recurrence in patients with HCC. Authors concluded that the NLR may be an efficient prognostic factor for HCC, but observations were performed for East Asian region populations where the incidence of HCC is higher [38].

Interestingly, Lee and Hong revealed a paucity of significant correlations between TINs and clinical outcomes in patients with early-stage HCC undergoing radical resection. Authors have suggested that TINs may fulfill an important prognostic role in the more advanced HCC stages [37].

### 3.2. Bone Marrow-Derived Cells (BMDCs)

It has been demonstrated that human hepatic cells in adults may originate from bone marrow stem cells or circulating stem cells, indicating that damaged liver tissue might be potentially replaced by stem cells-derived hepatocytes. Chronic inflammation could induce liver infiltration by hematopoietic stem cells [39]. The key pathogenetic mechanisms of HCC are associated with liver injury and inflammation, which leads to the recruiting of bone marrow-derived cells (BMDCs) to participate in liver repair [40]. There were studies that described inconsistent results regarding the impact of bone marrow-derived mesenchymal stromal cells on tumor growth and the metastatic potential of HCC [41,42,43,44,45,46]. Hence, it was speculated that BMDCs behavior was dependent on the tumor microenvironment [41]. Barone et al. (2014) investigated the entanglement of BMDCs in hepatocarcinogenesis using transgenic mice infected with HBV. In their experimental model, the authors revealed that BMDCs do not participate in tumorigenesis, but they infiltrate inflammatory liver areas in the vicinity of the HBsAg encoding gene (HBs-Eg)-positive cells. To follow the BMDCs’ fate, a transplantation of bone marrow cells acquired from wild-type (without a tendency towards cancer) male mice were implemented into female HBV transgenic mice of the same age developing hepatocarcinoma. Thereby, a Y-chromosome originating from the BMDCs was detected. The Y-chromosome-positive cells appeared to not be observed in the neoplastic liver nodules during the remarking time. Interestingly, authors observed the phenomenon of cell fusion of the BMDCs and hepatocyte lineage, revealing Y-chromosome/HBs-Eg/hepatocyte nuclear factor 1 (HNF1)-positive cells. A selection of healthy BMDCs from wild-type donors was marked and an evaluation of their capability for hepatocarcinogenesis depending on the HCC microenvironment was performed [47]. Conversely, Chen et al. by exploiting a diethylnitrosamine (DEN)-induced HCC model, demonstrated that BMDCs may be an origin of HCC. Wild-type male mice were exposed to DEN after bone marrow transplantation from matched, green fluorescent protein (GFP)-expressing, transgenic male mice. A large amount of HCC cells obtained from recipients appeared to be positive for GFP. The origin of the HCC cells from the transplanted BMDSs was further confirmed by single-cell sequencing. Furthermore, to seal previous findings, researchers transplanted female mice bone marrow into male mice and subsequently, they exposed recipients to DEN for 16 weeks. Interestingly, all examined HCC cells originated from female donors and did not embody the Y chromosome [40]. The distinct results of the above-mentioned studies may ensue from the onset of the actions of the tumorigenic factor, because the hepatitis B virus-induced nodule formation occurred before the bone marrow transplantation, while the DEN HCC induction took place after stem cell grafting. Interestingly, Mansour et al. (2022), in their murine model, observed that BMDC transplantation after HCC induction by DEN caused liver regeneration and ameliorated liver function [48]. Bayo et al. revealed that the migration of BMDCs towards the HCC milieu is conditioned on the production of IL-8/CXCL8, *growth-regulated oncogene (GRO)/CXCL 1-2-3*, and monocyte chemotactic protein-1 (MCP-1)/CCL2 by the HCC microenvironment; however, there was no impact on HCC aggressiveness. Furthermore, the secretion of chemokines by HCC-stimulated BMDCs was observed, which promotes the recruitment of cellular components such as endothelial cells, fibroblasts, and peripheral blood mononuclear cells into the tumor milieu [44]. Moreover, the migration of mesenchymal stromal cells towards HCC may be triggered by the autocrine motility factor produced by an HCC tumor [49]. The contemporary blockage of receptors for CXCL1, and CXCL2 and of the autocrine motility factor resulted in the inhibition of more than 60% of mesenchymal stromal cell migration towards HCC in the experimental model [50].

Owing to their ability to the specifically migrate towards HCC, the therapeutic potential of the BMDCs was explored, especially as cellular drugs or gene carriers [41,49,50,51,52]. A recent study by Yu et al. conducted on cell lines and mouse models, revealed that BMDCs may be donors of extracellular vesicle (EV)-encapsulated *microRNA-375,* which has been described as a cancer suppressor [53,54]. Researchers demonstrated that EV-derived *microRNA-375* inhibited HCC cells proliferation, enhanced tumor cell apoptosis in vitro, and repressed tumor growth in vivo. An investigation of the regulatory mechanism showed that *microRNA-375* up-regulation leads to decreased *HOXB3* gene expression with a further inhibition of the Wnt/β-catenin pathway, leading to a restraint of malignant HCC cell phenotypes [53].

### 3.3. Tumor-Associated Mast Cells (TAMCs)

The mast cells (MCs) content in the HCC tissue appeared to be increased in comparison to the pericarcinomatous hepatic tissue [55]. Furthermore, tumor-associated mast cells (TAMCs) may increase tumor angiogenesis [55,56,57]. As a proangiogenic factor released from mast cells, secretory granules were explored such as tryptase—a serin protease acting inter alia through protease-activated receptor-2 (PAR-2) [56,58,59]. Ammendola et al. (2016) demonstrated that MCs positive to tryptase (MCT^+^) and endothelial cells positive to PAR-2 are strongly associated with each other and with microvascular density formation in HCC, indicating that tryptase inhibition may hamper angiogenesis and it may be a therapeutic target for HCC [56]. Moreover, MCs positive to tryptase are the main source of IL-17 production in the HCC microenvironment. The higher content of intra-tumoral MCT^+^ and IL-17-producing cells predictably significantly decreased the overall survival (OS) of patients with HCC [60]. The peri-tumoral inflammatory microenvironment may be crucial for HCC recurrence after resection [61,62,63]. Ju et al. (2009) explored the importance of peri-tumoral MCs to HCC outcomes after curative resections. Increased peri-tumoral MC infiltration was associated with inferior clinical outcomes and an increased 5-year recurrence incidence rate. High peri-tumoral MC density was strongly related to an increased early (2-year) recurrence rate [61]. Interestingly, the recent study by Ali et al. (2024), investigating the usefulness of QuPath software(v0.5) for the pathological assessment of immunohistochemical stained HCC slides, revealed that the elevated area fraction (AF) of mast cells in the inner margins and peri-tumor areas was associated with longer, disease-free survival (DFS) rates, and an elevated AF of MCs in the inner margin was also associated with favorable overall survival (OS) rates. The AF of MCs was elevated in all evaluated regions of interest, including the tumor center, inner margin, outer margin, and peri-tumor area. The MCs were significantly more abundant in all regions of interest compared to natural killer (NK) cells and immature dendritic cells (iDCs). Researchers concluded that TAMCs in the HCC microenvironment contributed to an improved prognosis for patients with HCC who had undergone curative resection, indicating that, owing to the inclusion of only resectional cases, the study population was not representative of the general HCC population. The QuPath software appeared to be a relevant tool for whole-slide image (WSI) analysis, providing repeatable quantitative data in a digital HCC pathological analysis and yielding prognostic information for TAMCs in resectable HCC [64]. Zhang et al. (2021) created a prognostic model for genes associated with high and low infiltration of MCs in HCC. The authors divided MCs into high- and low-risk groups. Interestingly, TAMC’s content appeared to be inversely correlated with the risk score. In the study, HCC transcriptome data were acquired from The Cancer Genome Atlas (TCGA) and the model was verified by two independent datasets. Ultimately, owing to the cost reduction, the model used two genes (KIF2C and G6PD) that, based on the available literature, play a valid role in tumorigenesis [65].

### 3.4. Liver-Sinusoidal Endothelial Cells (LSECs)

Liver-sinusoidal endothelial cells (LSECs) remain a unique type of highly specialized vascular cells forming liver sinusoids, which create an interface between the flowing intestinal blood, hepatocytes, and hepatic stellate cells. LSECs are characterized by the highest permeability among all endothelial cells in a mammalian body [66,67]. It is currently well established that along with HCC progression, LSECs shed their specific markers, including the lymphatic vessel endothelial hyaluronan receptor-1 (LYVE-1), CD32b, stabilin-1, and stabilin-2 [66,68]. Concurrently, LSECs exhibit an increased expression of integrins, promoting HCC cell adhesion. LSECs intercellular adhesion molecule-1 (ICAM-1) expression appears to be decreased in HCC, causing a reduction of leukocyte infiltration and adhesion [66,67]. Furthermore, LSECs can also influence immune responses, entailing T-cell tolerance to HCC antigens [66,69]. One of the crucial processes underlying the progression from NAFLD or MAFLD through non-alcoholic steatohepatitis (NASH) to HCC is LSECs capillarization, which manifests through the loosening of the fenestrations and development of the basement membrane, which finally leads to pathological angiogenesis [70]. Manzi et al. (2016) revealed that the proliferation and migration of LSECs are regulated by galectin-1(Gal-1); Gal-1 is a β-galactoside binding protein that, through a glycosylation-dependent mechanism, plays a key role in vascular biology, and exhibits overexpression in the case of malignant tumors, affecting cell adhesion, and tumor-immune escape or tumor progression [71,72,73,74]. In the study employing human HCC cell lines and LSEC lines, named SK-HEP-1, it was observed that releasing Gal-1 from the HCC cells induced Gal-1 expression in the SK-HEP-1 cells and increased their proliferation and migration. Gal-1 also supported the adhesion of the HCC cells to LSECs. Furthermore, researchers demonstrated that TGF-β may significantly increase the release of Gal-1 from HCC cells and it may prompt Gal-1-dependent HCC cell adhesion to LSECs—which remains a key point of tumor metastasis [71]. Yu et al. proposed a new therapeutic strategy for HCC, based on a well-known drug—simvastatin—using nanotechnological to target LSECs [75]. It was demonstrated previously that statins may protect LSECs and alleviate endothelial–stellate cell activity, causing vasoprotective and anti-fibrotic effects. As a key mechanism for the aforementioned impressions, a transcription factor named Kruppel-like factor 2 (KLF2) was considered for induction in the LSECs [76,77]. Yu et al. (2022) emphasized that a poor prognosis in patients with HCC and fibrosis are associated with LSECs capillarization. Researchers showed that simvastatin increased the nitric oxide (NO) released from LSECs through the KLF2-endothelial nitric oxide synthase (eNOS) pathway that led to the deactivation of HSCs, alleviating the fibrotic processes associated with capillarization. Furthermore, simvastatin increased CXCL16 expression in LSECs, while CXCL16 remains a ligand for the CXCR6 receptor expressed by NK cells, causing the recruitment of natural killer (NK) cells and leading to an anti-tumor immune-mediating response. In the HCC murine model, simvastatin reduced LSEC capillarization and restrained tumor progression. Authors concluded that LSEC-targeting simvastatin administered through nano delivery appears as a promising therapeutic option for patients with HCC [75].

### 3.5. Tumor-Associated Macrophages (TAMs)

Monocytes and macrophages formation during cancer progression appears to be different than the origin of monocytes and tissue-resident macrophages in the non-tumorigenic state [78]. Cancer-related inflammation leads to the production of monocytes in the bone marrow through mediators such as IL-6, the granulocyte-macrophage colony stimulating factor (GM-CSF), or the granulocyte-colony stimulating factor (G-CSF) released by cancer cells, but splenic hematopoietic stem cells and progenitor cells are also involved in this process [78,79,80,81]. Formed monocytes infiltrate the tumor and in the tumor milieu they differentiate into tumor-associated macrophages (TAMs) [80]. Liver macrophages consist of resident Kupffer cells settled in the blood sinuses and monocyte-derived macrophages, which are recruited mainly during inflammation. HCC cells release various cytokines that cause the alteration of liver macrophages into TAMs, which reveal two distinct polarization states: M1 and M2, both interconvertible, depending on the tumor microenvironment [81,82,83]. M1-TAMs are classically activated through lipopolysaccharide, IFN-γ, tumor necrosis factor (TNF), GM-CSF, or Toll-like receptor (TLR) ligands, and exhibit pro-inflammatory and anti-cancer properties, releasing various cytokines [82]. M1-TAMs are frequently present in the peri-tumoral area, while M2-TAMs appear often in the HCC tissue [81]. M2-TAMs are alternatively activated through IL-4, IL-10, IL-13, TGF-β, colony-stimulating factor 1 (CSF-1), or prostaglandin E2 (PGE2), and, differently than M1-TAMs, reveal immunosuppressive properties, promoting tumor growth, angiogenesis, and metastasis [82,84]. In the process of metastasis, the involvement of M2-TAMs-secreted exosomes was considered, which contain inter alia miR-23a-3p that upgrade the epithelial–mesenchymal transition (EMT), increase vascular permeability, and promote angiogenesis [85]. In a recent study, Hao et al. (2022), using single-cell RNA-sequencing, demonstrated that the apolipoprotein C1 (APOC1) gene exhibits overexpression in TAMs derived from HCC tissues, and further authors revealed that APOC1 inhibition entails the transformation of the TAMs from an M2 phenotype to an M1 phenotype through a ferroptosis pathway as indicated in the altered genes expression which is distinctive for ferroptosis. APOC1 down-regulation induces the reduction of M2-TAMs, B cells, and CD4^+^ T cell levels in HCC, with simultaneous growth in M1-TAMs, NK cells, and CD8^+^ T cells restraining tumor growth and invasion. Moreover, APOC1 is inversely correlated with PD1/PD-L1 expression in HCC, leading to a boosted sensitivity during anti-PD1 therapy [86]. Tan et al. (2023) revealed that triggering a receptor expressed on myeloid cells 2 (TREM2)-positive TAMs negatively influenced CD8^+^ T cells infiltration of HCC after transarterial chemoembolization (TACE), indicating the possible cause of recurrence and HCC progression following TACE [87]. The TREM2 gene encodes the surface anti-inflammatory receptors distinctive for the myeloid lineage that negatively affects the Toll-like receptor (TLR)-mediated response and appears to be an important component of immune TME [88]. TREM2 overexpression at TAMs in HCC led to a poor prognosis, while TREM2 down-regulation enhanced CD8^+^ T cell infiltration, hindering tumor growth. It was also found that TREM2-positive TAMs produced a greater amount of galectin-1, which influences PD-L1 overexpression in tumor vessels, and attenuates CD8^+^ T cell infiltration. Authors concluded that TREM2 down-regulation may improve the results of anti-PD-L1 therapy in HCC by increasing the anti-tumor CD8^+^T cells’ action, and indicated that targeted anti-TREM2 immunotherapy may improve therapeutic outcomes after TACE, which itself remains a promising HCC treatment strategy [87].

### 3.6. Cancer-Associated Fibroblasts (CAFs)

The origin of cancer-associated fibroblasts (CAFs) in HCC remains unclear and currently available evidence shows that CAFs may emanate from HSCs, parenchymal cells that underwent an epithelial–mesenchymal transition, BMDCs, portal fibroblasts, or mesothelial cells. CAFs, by releasing various cytokines and factors negatively affect an HCC prognosis, increasing tumor growth, angiogenesis, and epithelial–mesenchymal transition, and alleviating HCC immune surveillance [89]. Activated CAFs produce extracellular matrix (ECM) components such as laminin, fibronectin, fibrillar collagen, and proteoglycans, affecting mechanically and biochemically the ECM properties, e.g., the stiffness that prompts HCC invasion or anti-tumor drug resistance [90,91,92]. Among CAFs releasing cytokines, CCL5 or CXCL11 exacerbate HCC malignancy and promote metastasis; further, proteins such as the hepatocyte growth factor (HGF) or follistatin-like protein 1 (FSTL-1) also reveal prometastatic abilities, while the blockage of the aforementioned factors improved HCC outcomes presented in the experimental models [90,93,94,95,96]. Also, CAFs inextricably create crosstalk with diverse immune cell types in the TME, causing the inter alia inhibition of NK cell and T-cell activity against HCC through various secreted mediators, e.g., PGE2, indoleamine 2,3-dioxygenase (IDO), or IL-6, and thereby lead to immunosuppression that favors HCC development and invasion [90]. Liu et al. (2023) recognized a tumor immune barrier (TIB) in an HCC tumor, located spatially near the tumor borders and consisting of CAFs, that secreted phosphoprotein 1 (SPP1)-positive macrophages and their products which may negatively affect immunotherapy efficacy. Delving into the molecular mechanisms, researchers showed that under hypoxic conditions, SPP1 expression on macrophages was increased and SPP1-positive macrophages affect CAFs leading to ECM remodeling through changes in the expression of collagen, matrix metalloproteinases, or chemokine genes, with a subsequent TIB forming that restrains immune cell infiltration into an HCC tumoral core. The experimental block of SPP1 significantly strengthened the efficiency of anti-PD1 immunotherapy, limited CAF infiltration, and exacerbated the infiltration and cytotoxic effect of CD8^+^ T cells in the mouse model of HCC, indicating the destruction of the TIB structures [97]. Interestingly, Eun et al. (2023) revealed that CAF-derived SPP1 overexpression is responsible for the resistance of HCC to tyrosine kinase inhibitors (TKI). An increased plasma SPP1 level in patients with advanced HCC treated with TKI was recognized as an independent predictor of unfavorable progression-free survival (PFS) and overall survival (OS) rates. Authors concluded that the CAF-derived SPP1 blockade might be a potential therapeutic target to alleviate TKI resistance in HCC treatment, whereas measurement of the plasma SPP1 level before TKI chemotherapy might predict responses to the planned treatment [98].

### 3.7. Lymphocytes

Tumor-infiltrating lymphocytes (TILs) in the HCC encompass the group of diverse immune cells such as CD8^+^ T cells, CD4^+^ T cells, Tregs, NK cells, or B cells that affect HCC progression through compound immunological mechanisms and interactions with various cells and TME [99]. The TILs density in tumor tissue provides a strong prognostic value; more abundant TIL infiltration and thereby high TIL density in the tumor area is associated with longer overall survival (OS) rates and disease-free survival (DFS) rates in patients with resectable HCC [100,101]. Overall, CD8^+^ T cells, CD4^+^ T cells, NK cells, and B cells reveal anti-tumor properties, while Tregs, similar to fibroblasts, M2 macrophages, or MDSCs, promote HCC progression [99]. CD8^+^ T lymphocytes remain the crucial effectors of immune cells and, through an interaction between their T cell receptor (TCR) and the major histocompatibility complex (MHC-I) antigen on tumor cells, they excrete granzyme B or perforin to exterminate HCC cells [99,102]. The density of high tissue-resident (CD103^+^) CD8^+^ T cells correlates significantly with favorable OS rates in patients with HCC. Furthermore, a higher CD103^+^CD8^+^T cells/total CD8^+^T cells ratio in HCC tissues links negatively with the advanced pathological HCC stages [102]. Immunotherapy remains an available option in the advanced HCC stages; however, therapeutic results are not always satisfactory [103]. It was recently demonstrated that low-dose radiotherapy (LDRT), in combination with anti-PD-L1 and anti-VEGFA (vascular endothelial growth factor A) therapy, ameliorates the anti-tumor response through the activation of CD8^+^ exhausted-like T cells (CD8^+^ Tex) in the tumoral core [104]. CD8^+^ Tex are formed gradually during HCC development through the decline in effector function or the ability to proliferate, which alleviates the anti-tumor immune response [104,105]. LDRT elicits inflammation in the tumor area and improves CD8^+^ Tex function and their cytolytic capacity. LDRT may also contribute to the reduction of the Treg/T-cell subset ratio. The mechanism of HCC sensitization to anti-PD-L1 and anti-VEGFA therapy by LDRT likely relies on the recruitment of stem-like progenitor CD8^+^ T(p)ex located in the draining lymph nodes and transported to the tumoral core through the chemokine (C-X-C motif) ligand 10/chemokine (C-X-C motif) receptor 3 (CXCL10/CXCR3) axis. CD8^+^ Tpex reveals the capability for regeneration, expansion, and differentiation [104]. In opposition to CD8^+^ T cells, regulatory T lymphocytes (Tregs) mainly provide an immunosuppressive tumor microenvironment; however, NK cells with an exhausted status that lost their cytotoxicity or a small number of memory B-cells in the activated state may also create an immunosuppressive HCC landscape. A gene set enrichment analysis (GSEA) revealed that Tregs paradoxically present an intensified metabolism in the HCC microenvironment through gluconeogenesis, glycolysis, the starch and sucrose metabolism, or the glutathione metabolism. An overexpression of inhibitory checkpoints, such as *CTLA4, TIGIT, TNFRSF4*, and *TNFRSF9* distinctive for Treg lymphocytes, may lead to the formation of a new targeting therapy restoring anti-cancer immunity [106]. Gu et al. (2022) revealed that lactate, being a plentiful tumor cell metabolite, influences Treg cells by promoting tumorigenesis via the lactylation of a membrane-organizing extension spike protein (MOESIN) at Lys 72, which enhances MOESIN interplay with the TGF-β receptor I and TGF-β signaling pathways in TME. An anti-lactate treatment strategy may improve anti-tumor outcomes in HCC, which has been explored in regard to anti-PD-1 therapy [107].

### 3.8. Hepatic Stellate Cells (HSCs)

**Figure 1:**
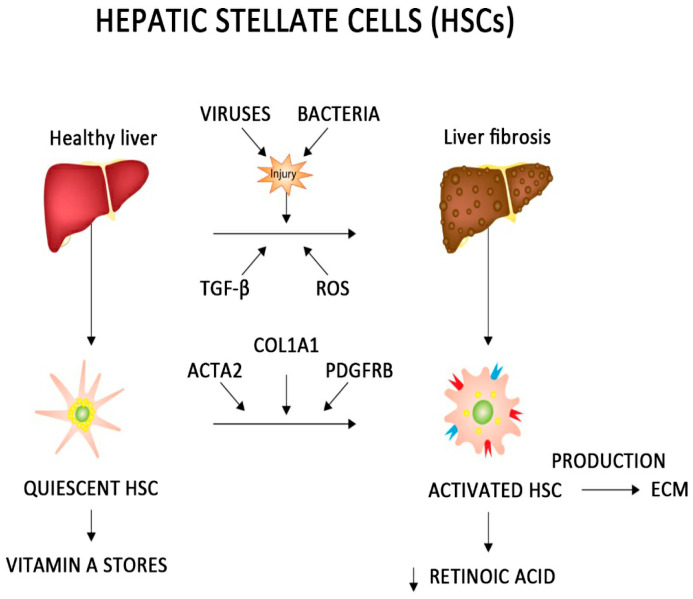
The role of hepatic stellate cells in the microenvironment of hepatocellular carcinoma. The figure was created with stock.adobe.com (accessed on 28 June 2024). Abbreviations: ECM—extracellular matrix, HSC—hepatic stellate cell, ROS—reactive oxygen species, and TGF β—transforming growth factor-β. ↓— reduction in concentration.

Hepatic stellate cells (HSCs) make up approximately 10 percent [108] of the total liver cell population. HSCs are mesenchymal cells, localized in the periaqueductal space (Disse space), and are the main fibrogenic cell type in a damaged liver [109]. At rest, hepatic stellate cells store more than 80% of the vitamin A accumulated in the liver as cytoplasmic lipid droplets [110,111]. Injury to the liver by, for example, viruses or bacteria, followed by the activation of the immune system, the secretion of cytokines, including the transforming growth factor-β (TGFβ), or the release of reactive oxygen species (ROS) are signals for the activation of hepatic stellate cells [112]. Activation is related to vitamin A metabolism and there is a decrease in the retinoic acid concentration within the stellate cells [113]. The activation of HSCs requires an induction of pro-fibrin genes such as *ACTA 2, COL1A1*, and *PDGFRB* from the resting state of the cell cycle to the DNA synthesis phase. This is regulated by E-type cyclins: cyclin E1 and cyclin E2 [114]. The activated HSCs produce an ECM at the site of the injury and initiate the process of fibrosis, and liver fibrosis, which is an important risk factor for the development and progression of HCC [115,116,117]. Increasing evidence suggests that activated hepatic stellate cells may play a key role in the release of cytokines including interleukin-6 (IL-6) and take an active role in tumorigenesis (Figure 1) [114,118].

### 3.9. Myeloid-Derived Suppressor Cells (MDSCs)

**Figure 2:**
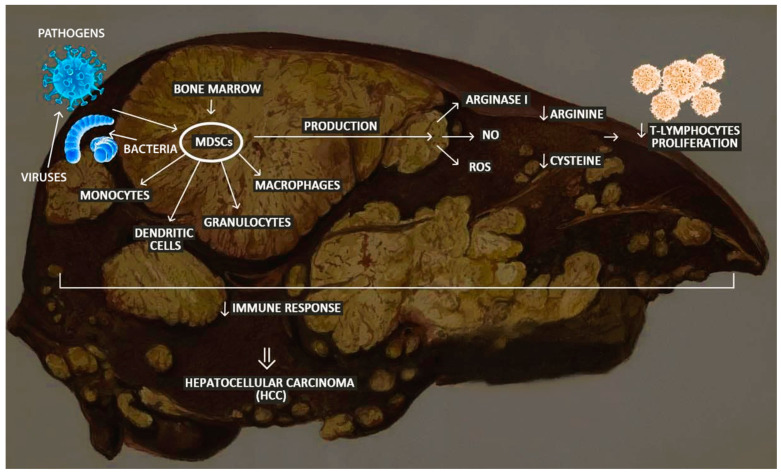
The role of myeloid-derived suppressor cells (MDSCs) in the microenvironment of the hepatocellular carcinoma. The figure was created with unsplash.com, stock.adobe.com, and pexels.com (accessed on 13/28 June 2024). Abbreviations: NO—nitric oxide, and ROS—reactive oxygen species. ↓—reduction.

Myeloid-derived suppressor cells (MDSCs) are immature populations of cells derived from the bone marrow [119]. They give rise to monocytes, dendritic cells, macrophages, and granulocytes. MDSCs recognize foreign pathogens, destroy them, and take control of the inflammatory response that takes place in the body [120]. Increasing evidence suggests that MDSCs are essential contributors to the development of HCC in a T-lymphocyte-dependent or independent manner [119]. MDSCs suppress an immune response through the production of arginase 1, nitric oxide, and reactive oxygen species or by secreting interleukin-10 (IL-10). MDSCs reduce the tissue availability of arginine and cysteine, which are essential for T-cell proliferation and impede cytotoxicity and the development of NK cells, resulting in tumor escape from immune surveillance [121]. A study conducted in a hospital in China, which ran from April 2017 to January 2019, involved 48 patients. The subjects were divided into three groups, patients diagnosed with HCC, 16 patients with a chronic HBV infection, and 21 healthy patients as a control. The relationship between MDSC, HCC, and markers of liver damage was then analyzed. It appeared that MDSCs have an important role in the process of escalation from an HBV infection to the development of cancer, as HBV induces the expansion of MDSCs which leads to T-cell depletion and the persistence of chronic inflammation. It was shown that an elevated percentage of MDSCs in the blood of cancer patients positively correlates with the progression of the cancer process and a worse prognosis [122]. It was also shown that MDSC levels were significantly higher in patients with HCC compared to a group of healthy individuals (Figure 2) [123].

### 3.10. Tumor-Associated Neutrophils (TANs)

Tumor-associated neutrophils (TANs) play a key role in cancer development, particularly the development of hepatocellular carcinoma. The presence of inflammation is an integral part of tumor development. A study conducted at Zhongshan Hospital investigated the effect of TANs on the development of HCC in vitro and in vivo. TANs were co-cultured with HCC cells. TANs were found to promote HCC growth using CCK8. Moreover, studies have shown that TANs secrete the bone morphogenetic protein BMP2 and transforming growth factor-β2 (TGF-β2), which promote the expression of *miR-301b-3p* in HCC cells [124]. TANs exhibit two phenotypes: anti-tumor N1 and pro-tumor N2. Tumor-related inflammatory stimulation shapes the neutrophil phenotype. Type I interferons support the production of TANs with anti-cancer activity (N1). ROS also influence the anti-tumor activity of neutrophils, leading to direct apoptosis of cancer cells. The pro-tumorigenic role of neutrophils results from the expression of the granulocyte-macrophage colony-stimulating factor (GM-CSF) and the tumor necrosis factor (TNF) in peri-tumoral tissues within HCC, enhancing the immunomodulatory effect of neutrophils and leading to the suppression of T lymphocytes [125,126]. It was shown that the accumulation of acetyl-Co-A induces an increase in the expression of the *CXCL1* gene, which recruits TANs and leads to the formation of extracellular neutrophil traps, which promote the metastasis of hepatocellular carcinoma [127]. Neutrophils also have a pro-angiogenic effect that may promote metastasis. Under the influence of CXCL1/MIP-*2*, they release the vascular endothelial growth factor A (VEGF-A), through which they modulates tumor growth. TANs also release matrix metalloproteinase-9 (MMP-9), which causes the release of VEGF from the ECM [128]. Moreover, CXCR2 is a receptor important for neutrophil recruitment. Research conducted by Jack Leslie et al. (2022) showed that therapy with a CXCR2 antagonist and anti-PD1 led to a reduction in the size of the HCC tumor, which proves the important role of neutrophilia in its development [129].

### 3.11. Tumor-Associated Endothelial Cells (TAECs)

Tumor-associated endothelial cells (TAECs) are components of the tumor microenvironment and play a role in angiogenesis [130]. HCC itself is a highly vascularized tumor that metastasizes through the hematogenous route. The functioning of endothelial cells depends mainly on the glycoinositol anchored in the cell—CD109. It occurs in the endothelial cells of the tumor and is non-cancerous in origin. It was shown that patients with low CD109 expression experienced more frequent tumor recurrences and shorter survival rates [131]. The neoplastic capillaries were severely twisted, immature, and had an irregular diameter. The tumor endothelial cells (TECs) tended to proliferate rapidly, and their basement membrane was discontinuous. In the microenvironment, TECs showed high instability and increased reactivity to proangiogenic factors [132]. TAECs are an important component of the tumor microenvironment. They display the following stem cell markers: CD90, Sca-1, MDR1 ALP, and Oct-4. They support tumor growth and metastasis [31]. They contain organelles of the tissue plasminogen activator (tPA) and organelles containing type 2 chemokines, which are responsible for the secretion of, among others, interleukin-6 (IL-6) and tPA [130]. TAECs support tumor growth and spread by creating blood vessels. The discontinuous basement membrane of TECs means that they can easily separate and metastasize in other places [133,134].

### 3.12. Extracellular Matrix (ECM)

The extracellular matrix (ECM) under normal conditions consists of collagens, glycosaminoglycans, and proteoglycans. It also includes cytokines, growth factors, proteases, and integrins [130]. ECM synthesis occurs through ALK4/*SMAD2/3* signaling activated by endoglin (CD105) [135,136]. ECM promotes the development of HCC through fibrosis [137]. The activation of stromal cells and excessive ECM deposition leads to increased integrin signaling and a stiffening of the liver tissue [138]. In a healthy liver, type IV collagen is the most abundant component of the ECM among the collagens [139]. Type I collagen plays a major role in the hepatocellular tumor microenvironment. Other ECM proteins are involved in the development of HCC, such as tenascin-C (TNC). There is an increase in TNC during chronic liver fibrosis [140].

### 3.13. Dendritic Cells (DCs)

Dendritic cells (DCs) induce an immune response. They are responsible for antigen presentation to T lymphocytes. A relationship between dendritic cells and the development of hepatocellular carcinoma has been demonstrated [141], and the number of tumor-associated DCs may serve as a prognostic factor in HCC, particularly in relation to tumor recurrence or the presence of metastases [142]. DCs have anti-cancer and pro-tumor effects. The first action results from the induction of an immune response involving T lymphocytes, and in the second scenario, a tolerance to tumor cells may develop by inhibiting receptor ligands, especially for PD1, which reduces the immune response [143]. CDs release cytokines, especially interleukin-12 (IL-12), which determine the polarization of emerging T helper lymphocytes. Therefore, DCs are becoming more and more widely used in the treatment of HCC. In a study conducted in China, mice in which HCC tumor cells were introduced were implanted with anti-PD-L1 antibodies and DCs on day seven, after the injection of tumor cells. The DCs were derived from each mouse’s bone marrow. The mice were then monitored to determine their length of survival, tumor volume was measured, histopathology was performed, and DC and lymphocyte levels were checked. The effectiveness of the DC vaccine in HCC therapy was based on the ability of the DCs to activate the T lymphocytes, while the use of anti-PD-L1 antibodies may have enhanced the anti-tumor response. Depending on the dose of both vaccines, the mouse survival time increased from 56 days to 86 days. Mice treated with dendritic cells in combination with anti-PD-L1 had a significantly reduced tumor volume compared to the control group. Moreover, a relationship was noticed: the higher the dose of anti-PD-L1, the smaller the tumor volume [144]. In the HCC microenvironment, DCs are often suppressed by various mechanisms, such as the production of immunosuppressive cytokines by tumor cells and the presence of other immunosuppressive cells, such as TAMs and MDSCs. These mechanisms lead to the impaired ability of DCs to effectively present antigens and activate T cells, which contributes to the tumor evading the immune response [145].

### 3.14. Cytokines

Inflammation is one of the factors leading to the development of hepatocellular carcinoma. In response to inflammation, cytokines mediate the migration of immune cells to damaged cells. Significantly high levels of cytokines, interleukin-5 (IL-5) and interleukin-6 (IL-6), were reported in the patients diagnosed with hepatocellular carcinoma compared to the healthy patients [146]. A pretreatment IL-6 level may be a prognostic indicator for patients with advanced HCC. At the Cancer Institute of China, Yi Dong Lin et al. conducted an approximately 2-year follow-up of 63 patients with advanced hepatocellular carcinoma. The relationship between circulating cytokine levels and prognosis was investigated in the patients with advanced hepatocellular carcinoma treated with radiotherapy in combination with monoclonal antibody therapy. Two groups of patients were distinguished—with low levels of IL-6 (10/47) and high levels (37/47). The risk of death in the low IL-6 group was significantly lower compared to the high group. AFP levels decreased by 58.5% in the low IL-6 group compared to the pretreatment levels, while they increased by 176.8% in the high IL-6 group [147]. The transforming growth factor β1 (TGF β1) plays an important role in liver fibrosis through, among other things, the activation of fibroblasts [148,149]. Moreover, the level of the transforming growth factor (TGF) increases significantly in a damaged liver, as a result of inflammation for example, and promotes the regeneration and dysplasia of hepatocytes. But it also inhibits the activation of leukocytes, which leads to the inhibition of the immune response and, paradoxically, may lead to the development of cancer [150]. Transforming growth factor α (TNF-α) is also believed to promote the development of hepatocellular carcinoma. In healthy liver cells, its expression is low, but when inflammation develops under the influence of other cytokines, the level of TNF-α increases significantly, resulting in the proliferation and dysplasia of hepatocytes [151]. A study by Jinget al. shown that inhibition of TNF-α may contribute to reducing the incidence of HCC. Moreover, TNF-α affects the activation of hepatic progenitor cells (HPCs), which are involved in liver repair and also contribute to carcinogenesis [152]. Serum interleukin-34 (IL-34) is correlated with liver inflammation and fibrosis in patients with chronic hepatitis B. Interleukin-32 (IL-32) is crucial in local invasion or distant metastasis perhaps by inhibiting the activity of NK cells against HCC, and NK cells have a protective function against liver fibrosis; there are studies on the use of invariant natural killer T in therapies for HCC [153,154,155]. Another research team reported a correlation between a circulating IL-32 and the area of VEGF staining in HCC tissues, suggesting that IL-32 may also enhance HCC development by increasing angiogenesis [153]. In a meta-analysis by Pan et al., CXCL8 concentrations were shown to be significantly higher in HCC patients [156]. A study by Wang et al. (2019) showed that CXCL 1/2/3/5/8 may serve as a therapeutic target for the treatment of HCC [157].

## 4. Conclusions

Inflammatory processes, cirrhosis, and liver fibrosis are significant in the development of HCC, and they are impacted by various variables including alcohol, HBV, HCV, and HDV infections, obesity, toxins, and mutations in the P53 suppressor gene. The above-mentioned cell types and environmental factors are important in the development of HCC and may be targeted for future treatment (Table 1). Understanding how they interact and how they affect the growth of a tumor may aid in the creation of HCC treatment plans that are more successful. To enhance the predictive and therapeutic biomarker landscapes and enhance the efficacy of HCC treatment, scientific investigations into the molecular mechanisms underlying the disease’s etiology are essential. Therapy that targets a particular cell type or cytokine may be introduced, which could greatly increase therapeutic efficacy and raise the chance of survival for HCC patients. Therefore, it is crucial to do more scientific research in this field to create cutting-edge, potent treatment plans in the fight against HCC.

## Figures and Tables

**Table 1:** Brief summary of the described immune and non-immune cell stroma as the microenvironment of HCC. Abbreviations: TINs—tumor-infiltrating neutrophils, HCC—hepatocellular carcinoma, BMDCs—bone marrow-derived cells, TAMCs—tumor-associated mast cells, MCs—mast cells, LSECs—liver-sinusoidal endothelial cells, TAMs—tumor-associated macrophages, CAFs—cancer-associated fibroblasts, TILs—tumor-infiltrating lymphocytes, NK—natural killer, HSCs—hepatic stellate cells, MDSCs—myeloid-derived suppressor cells, TANs—tumor-associated neutrophils, TAECs—tumor-associated endothelial cells, ECM—extracellular matrix, DCs—dendritic cells.

Component	Significance/Effect	Publication
TINs	There is a lack of significant correlation between TINs and clinical outcomes in patients with early-stage HCC. High baseline neutrophil-to-lymphocyte (NRL) ratio associate with poor prognosis in patients with HCC.	[37,38]
BMDCs	Liver injury and inflammation preceding HCC development recruit BMDCs. Damaged liver cells may be replaced by BMDCs and BMDCs-derived hepatocytes may participate in hepatocarcinogenesis. Specific migration of BMDCs towards HCC cells may be exploit clinically to use BMDCs as cellular drugs or gene carriers.	[39,40,47,52,53]
TAMCs	Through tryptase releasing, TAMCs enhance tumor angiogenesis. High peritumoral MCs infiltration is associated with poor clinical outcomes and higher recurrence rate after HCC resection.	[56,57,58,61]
LSECs	LSECs promote HCC cells adhesion through increased expression of integrins, reduce leukocyte infiltration and adhesion, or cause T-cell tolerance to HCC antigens that leads to HCC progression. LSECs capillarization leads to pathological angiogenesis in HCC.	[66,67,69,70,71]
TAMs	Cancer-related inflammation recruit monocytes that infiltrate HCC milieu and differentiate into TAMs. In HCC milieu TAMs reveal two interconvertible polarization states: M1 exhibiting anti-tumor properties and M2 promoting tumor growth, angiogenesis and metastasis.	[78,80,81,82,84]
CAFs	CAFs release various cytokines in HCC milieu causing HCC progression and alleviating HCC immune surveillance. They produce extracellular matrix components prompting HCC invasion and causing anti-tumor drug resistance.	[89,90,91,92]
TILs	CD8+ T cells, CD4+ T cells, NK, and B cells manifest anti-tumor properties, while Tregs cause HCC progression. Increased TILs infiltration in the HCC tumor area associates with longer overall survival and disease-free survival in patients with resectable HCC.	[99,100,101]
HSCs	Activated HSCs produce extracellular matrix (ECM) at the site of injury and initiate the fibrosis process. Liver fibrosis is an important risk factor for the development and progression of HC.	[115,116,117]
MDSCs	MDSCs contribute to tumour escape from immune surveillance by reducing T-lymphocyte proliferation and inhibiting NK cell growth. They suppress the immune response by producing arginase 1, nitric oxide and producing reactive oxygen species or secreting interleukin-10 (IL-10).	[119,121]
TANs	Tumour-associated neutrophils show two phenotypes: anti-tumour N1 and pro-tumour N2. Neutrophils with the N2 phenotype enhance the immunomodulatory effect of HCC. The pro-tumour role of neutrophils is due to the expression of granulocyte-macrophage colony-stimulating factor (GM-CSF) and tumour necrosis factor (TNF) in peritumour tissues within HCC. Using CCK8, they promote HCC growth.	[124,125,126]
TAECs	TAECs exhibit the following stem cell markers: CD90, Sca-1, MDR1 ALP and Oct-4. They promote tumor growth and spread by, among other things, promoting blood vessel formation.	[31,133,134]
ECM	ECM promotes the development of HCC through fibrosis.	[137]
DCs	DCs increase the body’s tolerance to the presence of cancer cells by inhibiting receptor ligands, particularly for PD1, resulting in a reduced immune response.	[141]
Cytokines	Inflammation is one of the factors leading to the development and progression of HCC. Patients diagnosed with HCC had significantly higher levels of proinflammatory cytokines, including interleukin IL-6 (IL-6) and interleukin IL-5 (IL-5).	[146]

## Data Availability

Not applicable.

## Abbreviations

AFP—alpha-fetoprotein; BMDC—bone marrow-derived cells; BMP2—bone morphogenetic protein 2; CAF—cancer-associated fibroblasts; CD 105—endoglin; CD 109—glycoinositol; DC—dendritic cell; ECM—extracellular matrix; GM-CSF—granulocyte-macrophage colony-stimulating factor; HCC—hepatocellular carcinoma; HPC—hepatic progenitor cells; HSC—hepatic stellate cell; IL-5—interleukin-5; IL-6—interleukin-6; IL-10—interleukin-10; IL-12—interleukin-12; IL-32—interleukin-32; IL-34—interleukin-34; LSEC—liver-sinusoidal endothelial cell; MDSC—myeloid-derived suppressor cell; MMP-9—matrix metalloproteinase-9; NAFLD—non-alcoholic fatty liver disease; NASH—non-alcoholic steatohepatitis; NK—natural killer; PD-L1—programmed death-ligand 1; ROS—reactive oxygen species; TAEC—tumor-associated endothelial cell; TAMC—tumor-associated mast cell; TAN—tumor-associated neutrophil; TGF—transforming growth factor; TGFβ—transforming growth factor β; TGFβ1—transforming growth factor β1; TGFβ2—transforming growth factor β2; TIN—tumor-infiltrating neutrophils; TNC—tenascin-C; TNF—tumor necrosis factor; TNF-α—transforming growth factor α; tPA—tissue plasminogen activator; VEGF-A—vascular endothelial growth factor A.

## References

[1] Absolute Numbers, Incidence, Both Sexes, in 2022. https://gco.iarc.fr/today/en/dataviz/pie?mode=cancer&group_populations=1&cancers=39.

[2] Absolute Numbers, Incidence, Both Sexes, in 2022. https://gco.iarc.fr/today/en/dataviz/pie?mode=population&group_populations=0&cancers=11.

[3] Toh M.R., Wong E.Y.T., Wong S.H., Ng A.W.T., Loo L.H., Chow P.K., Ngeow J. (2023). Global Epidemiology and Genetics of Hepatocellular Carcinoma. Gastroenterology.

[4] Bertocchi A., Carloni S., Ravenda P.S., Bertalot G., Spadoni I., Lo Cascio A., Gandini S., Lizier M., Braga D., Asnicar F. (2021). Gut vascular barrier impairment leads to intestinal bacteria dissemination and colorectal cancer metastasis to liver. Cancer Cell.

[5] Sia D., Villanueva A., Friedman S.L., Llovet J.M. (2017). Liver Cancer Cell of Origin, Molecular Class, and Effects on Patient Prognosis. Gastroenterology.

[6] Aravalli R.N. (2013). Role of innate immunity in the development of hepatocellular carcinoma. World J. Gastroenterol..

[7] Ogunwobi O.O., Harricharran T., Huaman J., Galuza A., Odumuwagun O., Tan Y., Ma G.X., Nguyen M.T. (2019). Mechanisms of hepatocellular carcinoma progression. World J Gastroenterol..

[8] Hepatocellular Carcinoma. https://www.ncbi.nlm.nih.gov/books/NBK559177/.

[9] Ayuso C., Rimola J., Vilana R., Burrel M., Darnell A., García-Criado Á., Bianchi L., Belmonte E., Caparroz C., Barrufet M. (2018). Diagnosis and staging of hepatocellular carcinoma (HCC): Current guidelines. Eur. J. Radiol..

[10] Kim E., Viatour P. (2020). Hepatocellular carcinoma: Old friends and new tricks. Exp. Mol. Med..

[11] Russo F.P., Imondi A., Lynch E.N., Farinati F. (2018). When and how should we perform a biopsy for HCC in patients with liver cirrhosis in 2018? A review. Dig Liver Dis..

[12] Mehta N., Bhangui P., Yao F.Y., Mazzaferro V., Toso C., Akamatsu N., Durand F., Ijzermans J., Polak W., Zheng S. (2020). Liver Transplantation for Hepatocellular Carcinoma. Working Group Report from the ILTS Transplant Oncology Consensus Conference. Transplantation.

[13] Raoul J.L., Forner A., Bolondi L., Cheung T.T., Kloeckner R., de Baere T. (2019). Updated use of TACE for hepatocellular carcinoma treatment: How and when to use it based on clinical evidence. Cancer Treat. Rev..

[14] Zhu A.X., Abbas A.R., de Galarreta M.R., Guan Y., Lu S., Koeppen H., Zhang W., Hsu C.H., He A.R., Ryoo B.Y. (2022). Molecular correlates of clinical response and resistance to atezolizumab in combination with bevacizumab in advanced hepatocellular carcinoma. Nat. Med..

[15] Huang D.Q., Mathurin P., Cortez-Pinto H., Loomba R. (2023). Global epidemiology of alcohol-associated cirrhosis and HCC: Trends, projections and risk factors. Nat. Rev. Gastroenterol. Hepatol..

[16] Taniai M. (2020). Alcohol and hepatocarcinogenesis. Clin. Mol. Hepatol..

[17] D’souza S., Lau K.C., Coffin C.S., Patel T.R. (2020). Molecular mechanisms of viral hepatitis induced hepatocellular carcinoma. World J. Gastroenterol..

[18] McGlynn K.A., Petrick J.L., El-Serag H.B. (2021). Epidemiology of Hepatocellular Carcinoma. Hepatology.

[19] Chen Y. (2019). Tian ZHBV-Induced Immune Imbalance in the Development of HCC. Front. Immunol..

[20] Jiang Y., Han Q., Zhao H., Zhang J. (2021). The Mechanisms of HBV-Induced Hepatocellular Carcinoma. J. Hepatocell. Carcinoma.

[21] Anstee Q.M., Reeves H.L., Kotsiliti E., Govaere O., Heikenwalder M. (2019). From NASH to HCC: Current concepts and future challenges. Nat. Rev. Gastroenterol. Hepatol..

[22] Eslam M., Sanyal A.J., George J., International Consensus Panel (2020). MAFLD: A Consensus-Driven Proposed Nomenclature for Metabolic Associated Fatty Liver Disease. Gastroenterology.

[23] Myers S., Neyroud-Caspar I., Spahr L., Gkouvatsos K., Fournier E., Giostra E., Magini G., Frossard J.L., Bascaron M.E., Vernaz N. (2021). NAFLD and MAFLD as emerging causes of HCC: A populational study. JHEP Rep..

[24] Gofton C., Upendran Y., Zheng M.H., George J. (2023). MAFLD: How is it different from NAFLD?. Clin. Mol. Hepatol..

[25] Rushing B.R., Selim M.I. (2019). Aflatoxin B1: A review on metabolism, toxicity, occurrence in food, occupational exposure, and detoxification methods. Food Chem. Toxicol..

[26] Pickova D., Ostry V., Toman J., Malir F. (2021). Aflatoxins: History, Significant Milestones, Recent Data on Their Toxicity and Ways to Mitigation. Toxins.

[27] Surial B., Ramírez Mena A., Roumet M., Limacher A., Smit C., Leleux O., Mocroft A., van der Valk M., Bonnet F., Peters L. (2023). External validation of the PAGE-B score for HCC risk prediction in people living with HIV/HBV coinfection. J. Hepatol..

[28] Okeke E., Mark Davwar P., Mullen B., Duguru M., Agbaji O., Sagay A., Murphy R., Hawkins C. (2021). The impact of HIV on hepatocellular cancer survival in Nigeria. Trop. Med. Int. Health.

[29] SenGupta S., Hein L.E., Parent C.A. (2021). The Recruitment of Neutrophils to the Tumor Microenvironment Is Regulated by Multiple Mediators. Front. Immunol..

[30] Antuamwine B.B., Bosnjakovic R., Hofmann-Vega F., Wang X., Theodosiou T., Iliopoulos I., Brandau T. (2023). N1 versus N2 and PMN-MDSC: A critical appraisal of current concepts on tumor-associated neutrophils and new directions for human oncology. Immunol. Rev..

[31] Wang J., Bo X., Suo T., Liu H., Ni X., Shen S., Li M., Xu J., Liu H., Wang Y. (2018). Tumor-infiltrating neutrophils predict prognosis and adjuvant chemotherapeutic benefit in patients with biliary cancer. Cancer Sci..

[32] Wang J., Jia Y., Wang N., Zhang X., Tan B., Zhang G., Cheng Y. (2014). The clinical significance of tumor-infiltrating neutrophils and neutrophil-to-CD8+ lymphocyte ratio in patients with resectable esophageal squamous cell carcinoma. J. Transl. Med..

[33] Zhang H., Liu H., Shen Z., Lin C., Wang X., Qin J., Qin X., Xu J., Sun Y. (2018). Tumor-infiltrating Neutrophils is Prognostic and Predictive for Postoperative Adjuvant Chemotherapy Benefit in Patients With Gastric Cancer. Ann. Surg..

[34] Xiao Y., Cong M., Li J., He D., Wu Q., Tian P., Wang Y., Yang S., Liang C., Liang Y. (2021). Cathepsin C promotes breast cancer lung metastasis by modulating neutrophil infiltration and neutrophil extracellular trap formation. Cancer Cell.

[35] Zhao Y., Liu Z., Liu G., Zhang Y., Liu S., Gan D., Chang W., Peng X., Sung E., Gilbert K. (2023). Neutrophils resist ferroptosis and promote breast cancer metastasis through aconitate decarboxylase 1. Cell Metab..

[36] Wang J., Liu L., Bai Q., Ou C., Xiong Y., Qu Y., Wang Z., Xia Y., Guo J., Xu J. (2018). Tumor-infiltrating neutrophils predict therapeutic benefit of tyrosine kinase inhibitors in metastatic renal cell carcinoma. Oncoimmunology.

[37] Lee J.H., Hong Y.M. (2024). The relationship between tumor-infiltrating neutrophils and clinical outcomes in patients with resectable hepatocellular carcinoma. BMC Cancer.

[38] Lin S., Hu S., Ran Y., Wu F. (2021). Neutrophil-to-lymphocyte ratio predicts prognosis of patients with hepatocellular carcinoma: A systematic review and meta-analysis. Transl. Cancer Res..

[39] Alison M.R., Poulsom R., Jeffery R., Dhillon A.P., Quaglia A., Jacob J., Novelli M., Prentice G., Williamson J., Wright N.A. (2000). Hepatocytes from non-hepatic adult stem cells. Nature.

[40] Chen L., Yi X., Guo P., Guo H., Chen Z., Hou C., Qi L., Wang Y., Li C., Liu P. (2020). The role of bone marrow-derived cells in the origin of liver cancer revealed by single-cell sequencing. Cancer Biol. Med..

[41] Bayo J., Marrodán M., Aquino J.B., Silva M., García M.G., Mazzolini G. (2014). The therapeutic potential of bone marrow-derived mesenchymal stromal cells on hepatocellular carcinoma. Liver Int..

[42] Chen X., Lin X., Zhao J., Shi W., Zhang H., Wang Y., Kan B., Du L., Wang B., Wei Y. (2008). A tumor-selective biotherapy with prolonged impact on established metastases based on cytokine gene-engineered MSCs. Mol. Ther..

[43] Lu Y.R., Yuan Y., Wang X.J., Wei L.L., Chen Y.N., Cong C., Li S.F., Long D., Tan W.D., Mao Y.Q. (2008). The growth inhibitory effect of mesenchymal stem cells on tumor cells in vitro and in vivo. Cancer Biol. Ther..

[44] Qiao L., Xu Z., Zhao T., Zhao Z., Shi M., Zhao R.C., Ye L., Zhang X. (2008). Suppression of tumorigenesis by human mesenchymal stem cells in a hepatoma model. Cell Res..

[45] Li G.C., Ye Q.H., Xue Y.H., Sun H.J., Zhou H.J., Ren N., Jia H.L., Shi J., Wu J.C., Dai C. (2010). Human mesenchymal stem cells inhibit metastasis of a hepatocellular carcinoma model using the MHCC97-H cell line. Cancer Sci..

[46] Niess H., Bao Q., Conrad C., Zischek C., Notohamiprodjo M., Schwab F., Schwarz B., Huss R., Jauch K.W., Nelson P.J. (2011). Selective targeting of genetically engineered mesenchymal stem cells to tumor stroma microenvironments using tissue-specific suicide gene expression suppresses growth of hepatocellular carcinoma. Ann. Surg..

[47] Barone M., Scavo M.P., Maiorano E., Di Leo A., Francavilla A. (2014). Bone marrow-derived stem cells and hepatocarcinogenesis in hepatitis B virus transgenic mice. Dig. Liver Dis..

[48] Mansour W., Kamel M., Elzayat E., Atta S., Kamel M., Mahmood D., Sayed H.A.E.F., Hussein T., Saber S. (2022). Therapeutic Role of Bone Marrow-Derived Mesenchymal Stem Cells in Controlling Prognosis of Hepatocellular Carcinoma in a Murine Model. Exp. Clin. Transplant..

[49] Bayo J., Fiore E., Aquino J.B. (2014). Increased migration of human mesenchymal stromal cells by autocrine motility factor (AMF) resulted in enhanced recruitment towards hepatocellular carcinoma. PLoS ONE.

[50] Bayo J., Real A., Fiore E.J., Malvicini M., Sganga L., Bolontrade M., Andriani O., Bizama C., Fresno C., Podhajcer O. (2016). IL-8, GRO and MCP-1 produced by hepatocellular carcinoma microenvironment determine the migratory capacity of human bone marrow-derived mesenchymal stromal cells without affecting tumor aggressive-ness. Oncotarget..

[51] Zhang B., Shan H., Li D., Li Z.R., Zhu K.S., Jiang Z.B. (2012). The inhibitory effect of MSCs expressing TRAIL as a cellular delivery vehicle in combination with cisplatin on hepatocellular carcinoma. Cancer Biol. Ther..

[52] Knoop K., Kolokythas M., Klutz K., Willhauck M.J., Wunderlich N., Draganovici D., Zach C., Gildehaus F.J., Böning G., Göke B. (2011). Image-guided, tumor stroma-targeted 131I therapy of hepatocellular cancer after systemic mesenchymal stem cell-mediated NIS gene delivery. Mol. Ther..

[53] Yu Z., Liu J., Fan Q., Yu J., Ren X., Wang X. (2022). Extracellular Vesicle-Encapsulated MicroRNA-375 from Bone Marrow-Derived Mesenchymal Stem Cells Inhibits Hepatocellular Carcinoma Progression through Regulating HOXB3-Mediated Wnt/*β*-Catenin Pathway. Anal. Cell Pathol..

[54] Wei J., Lu Y., Wang R., Xu X., Liu Q., He S., Pan H., Liu X., Yuan B., Ding Y. (2021). MicroRNA-375: Potential cancer suppressor and therapeutic drug. Biosci. Rep..

[55] Peng S.H., Deng H., Yang J.F., Xie P.P., Li C., Li H., Feng D.Y. (2005). Significance and relationship between infiltrating inflammatory cell and tumor angiogenesis in hepatocellular carcinoma tissues. World J. Gastroenterol..

[56] Ammendola M., Sacco R., Sammarco G., Piardi T., Zuccalà V., Patruno R., Zullo A., Zizzo N., Nardo B., Marech I. (2016). Mast cells positive to tryptase, endothelial cells positive to protease-activated receptor-2, and microvascular density correlate among themselves in hepatocellular carcinoma patients who have undergone surgery. Onco Targets Ther..

[57] Grizzi F., Franceschini B., Chiriva-Internati M., Liu Y., Hermonat P.L., Dioguardi N. (2003). Mast cells and human hepatocellular carcinoma. World J. Gastroenterol..

[58] Ammendola M., Sacco R., Sammarco G., Donato G., Montemurro S., Ruggieri E., Patruno R., Marech I., Cariello M., Vacca A. (2014). Correlation between serum tryptase, mast cells positive to tryptase and microvascular density in colo-rectal cancer patients: Possible biological-clinical significance. PLoS ONE.

[59] Ammendola M., Sacco R., Sammarco G., Donato G., Zuccalà V., Romano R., Luposella M., Patruno R., Vallicelli C., Verdecchia G.M. (2013). Mast Cells Positive to Tryptase and c-Kit Receptor Expressing Cells Correlates with Angiogenesis in Gastric Cancer Patients Surgically Treated. Gastroenterol. Res. Pract..

[60] Tu J.F., Pan H.Y., Ying X.H., Lou J., Ji J.S., Zou H. (2016). Mast Cells Comprise the Major of Interleukin 17-Producing Cells and Predict a Poor Prognosis in Hepatocellular Carcinoma. Medicine.

[61] Ju M.J., Qiu S.J., Gao Q., Fan J., Cai M.Y., Li Y.W., Tang Z.Y. (2009). Combination of peritumoral mast cells and T-regulatory cells predicts prognosis of hepatocellular carcinoma. Cancer Sci..

[62] Zhu X.D., Zhang J.B., Zhuang P.Y., Zhu H.G., Zhang W., Xiong Y.Q., Wu W.Z., Wang L., Tang Z.Y., Sun H.C. (2008). High expression of macrophage colony-stimulating factor in peritumoral liver tissue is associated with poor survival after curative resection of hepatocellular carcinoma. J. Clin. Oncol..

[63] Hoshida Y., Villanueva A., Kobayashi M., Peix J., Chiang D.Y., Camargo A., Gupta S., Moore J., Wrobel M.J., Lerner J. (2008). Gene expression in fixed tissues and outcome in hepatocellular carcinoma. N. Engl. J. Med..

[64] Ali E., Červenková L., Pálek R., Ambrozkiewicz F., Pavlov S., Ye W., Hošek P., Daum O., Liška V., Hemminki K. (2024). Mast Cells in the Microenvironment of Hepatocellular Carcinoma Confer Favorable Prognosis: A Retrospective Study using QuPath Image Analysis Software. J. Vis. Exp..

[65] Zhang H., Sun L., Hu X. (2021). Mast Cells Resting-Related Prognostic Signature in Hepatocellular Carcinoma. J. Oncol..

[66] Hammoutene A., Rautou P.E. (2019). Role of liver sinusoidal endothelial cells in non-alcoholic fatty liver disease. J. Hepatol..

[67] Poisson J., Lemoinne S., Boulanger C., Durand F., Moreau R., Valla D., Rautou P.E. (2017). Liver sinusoidal endothelial cells: Physiology and role in liver diseases. J. Hepatol..

[68] Géraud C., Mogler C., Runge A., Evdokimov K., Lu S., Schledzewski K., Arnold B., Hämmerling G., Koch P.S., Breuhahn K. (2013). Endothelial transdifferentiation in hepatocellular carcinoma: Loss of Stabilin-2 expression in peri-tumourous liver correlates with increased survival. Liver Int..

[69] Höchst B., Schildberg F.A., Böttcher J., Metzger C., Huss S., Türler A., Overhaus M., Knoblich A., Schneider B., Pantelis D. (2012). Liver sinusoidal endothelial cells contribute to CD8 T cell tolerance toward circulating carcinoembryonic antigen in mice. Hepatology.

[70] Velliou R.I., Legaki A.I., Nikolakopoulou P., Vlachogiannis N.I., Chatzigeorgiou A. (2023). Liver endothelial cells in NAFLD and transition to NASH and HCC. Cell Mol. Life Sci..

[71] Manzi M., Bacigalupo M.L., Carabias P., Elola M., Wolfenstein-Todel C., Rabinovich G.A., Espelt M.V., Troncoso M.F. (2016). Galectin-1 Controls the Proliferation and Migration of Liver Sinusoidal Endothelial Cells and Their Interaction With Hepatocarcinoma Cells. J. Cell Physiol..

[72] Wu M.H., Ying N.W., Hong T.M., Chiang W.F., Lin Y.T., Chen Y.L. (2014). Galectin-1 induces vascular permeability through the neuropilin-1/vascular endothelial growth factor receptor-1 complex. Angiogenesis.

[73] Demydenko D., Berest I. (2009). Expression of galectin-1 in malignant tumors. Exp. Oncol..

[74] Ito K., Stannard K., Gabutero E., Clark A.M., Neo S.Y., Onturk S., Blanchard H., Ralph S.J. (2012). Galectin-1 as a potent target for cancer therapy: Role in the tumor microenvironment. Cancer Metastasis Rev..

[75] Yu Z., Guo J., Liu Y., Wang M., Liu Z., Gao Y., Huang L. (2022). Nano delivery of simvastatin targets liver sinusoidal endothelial cells to remodel tumor microenvironment for hepatocellular carcinoma. J. Nanobiotechnol..

[76] Marrone G., Russo L., Rosado E., Hide D., García-Cardeña G., García-Pagán J.C., Bosch J., Gracia-Sancho J. (2013). The transcription factor KLF2 mediates hepatic endothelial protection and paracrine endothelial-stellate cell deactivation induced by statins. J. Hepatol..

[77] Marrone G., Maeso-Díaz R., García-Cardena G., Abraldes J.G., García-Pagán J.C., Bosch J., Gracia-Sancho J. (2015). KLF2 exerts antifibrotic and vasoprotective effects in cirrhotic rat livers: Behind the molecular mechanisms of statins. Gut.

[78] Arvanitakis K., Koletsa T., Mitroulis I., Germanidis G. (2022). Tumor-Associated Macrophages in Hepatocellular Carcinoma Pathogenesis, Prognosis and Therapy. Cancers.

[79] McAllister S.S., Weinberg R.A. (2014). The tumour-induced systemic environment as a critical regulator of cancer progression and metastasis. Nat. Cell Biol..

[80] Cortez-Retamozo V., Etzrodt M., Newton A., Rauch P.J., Chudnovskiy A., Berger C., Ryan R.J., Iwamoto Y., Marinelli B., Gorbatov R. (2012). Origins of tumor-associated macrophages and neutrophils. Proc. Natl. Acad. Sci. USA.

[81] Yuan Y., Wu D., Li J., Huang D., Zhao Y., Gao T., Zhuang Z., Cui Y., Zheng D.Y., Tang Y. (2023). Mechanisms of tumor-associated macrophages affecting the progression of hepatocellular carcinoma. Front. Pharmacol..

[82] Cheng K., Cai N., Zhu J., Yang X., Liang H., Zhang W. (2022). Tumor-associated macrophages in liver cancer: From mechanisms to therapy. Cancer Commun..

[83] Rabold K., Netea M.G., Adema G.J., Netea-Maier R.T. (2017). Cellular metabolism of tumor-associated macrophages—Functional impact and consequences. FEBS Lett..

[84] Mantovani A., Marchesi F., Malesci A., Laghi L., Allavena P. (2017). Tumour-associated macrophages as treatment targets in oncology. Nat. Rev. Clin. Oncol..

[85] Lu Y., Han G., Zhang Y., Zhang L., Li Z., Wang Q., Chen Z., Wang X., Wu J. (2023). M2 macrophage-secreted exosomes promote metastasis and increase vascular permeability in hepatocellular carcinoma. Cell Commun. Signal..

[86] Hao X., Zheng Z., Liu H., Zhang Y., Kang J., Kong X., Rong D., Sun G., Sun G., Liu L. (2022). Inhibition of APOC1 promotes the transformation of M2 into M1 macrophages via the ferroptosis pathway and enhances anti-PD1 immunotherapy in hepatocellular carcinoma based on single-cell RNA sequencing. Redox Biol..

[87] Tan J., Fan W., Liu T., Zhu B., Liu Y., Wang S., Wu J., Liu J., Zou F., Wei J. (2023). TREM2^+^macrophages suppress CD8^+^T-cell infiltration after transarterial chemoembolisation in hepatocellular carcinoma. J. Hepatol..

[88] Zhou L., Wang M., Guo H., Hou J., Zhang Y., Li M., Wu X., Chen X., Wang L. (2022). Integrated Analysis Highlights the Immunosuppressive Role of TREM2^+^Macrophages in Hepatocellular Carcinoma. Front. Immunol..

[89] Baglieri J., Brenner D.A., Kisseleva T. (2019). The Role of Fibrosis and Liver-Associated Fibroblasts in the Pathogenesis of Hepatocellular Carcinoma. Int. J. Mol. Sci..

[90] Ying F., Chan M.S.M., Lee T.K.W. (2023). Cancer-Associated Fibroblasts in Hepatocellular Carcinoma and Cholangiocarcinoma. Cell Mol. Gastroenterol. Hepatol..

[91] Levental K.R., Yu H., Kass L., Lakins J.N., Egeblad M., Erler J.T., Fong S.F., Csiszar K., Giaccia A., Weninger W. (2009). Matrix crosslinking forces tumor progression by enhancing integrin signaling. Cell.

[92] Schrader J., Gordon-Walker T.T., Aucott R.L., Deemter M., Quaas A., Walsh S., Benten D., Forbes S.J., Wells R.G., Iredale J.P. (2011). Matrix stiffness modulates proliferation, chemotherapeutic response, and dormancy in hepatocellular carcinoma cells. Hepatology.

[93] Xu H., Zhao J., Li J., Zhu Z., Cui Z., Liu R., Lu R., Yao Z., Xu Q. (2022). Cancer associated fibroblast-derived CCL5 promotes hepatocellular carcinoma metastasis through activating HIF1α/ZEB1 axis. Cell Death Dis..

[94] Liu G., Sun J., Yang Z.F., Zhou C., Zhou P.Y., Guan R.Y., Sun B.Y., Wang Z.T., Zhou J., Fan J. (2021). Cancer-associated fibroblast-derived CXCL11 modulates hepatocellular carcinoma cell migration and tumor metastasis through the circUBAP2/miR-4756/IFIT1/3 axis. Cell Death Dis..

[95] Lau E.Y., Lo J., Cheng B.Y., Ma M.K., Lee J.M., Ng J.K., Chai S., Lin C.H., Tsang S.Y., Ma S. (2016). Cancer-Associated Fibroblasts Regulate Tumor-Initiating Cell Plasticity in Hepatocellular Carcinoma through c-Met/FRA1/HEY1 Signaling. Cell Rep..

[96] Loh J.J., Li T.W., Zhou L., Wong T.L., Liu X., Ma V.W.S., Lo C.M., Man K., Lee T.K., Ning W. (2021). FSTL1 Secreted by Activated Fibroblasts Promotes Hepatocellular Carcinoma Metastasis and Stemness. Cancer Res..

[97] Liu Y., Xun Z., Ma K., Liang S., Li X., Zhou S., Sun L., Liu Y., Du Y., Guo X. (2023). Identification of a tumour immune barrier in the HCC microenvironment that determines the efficacy of immunotherapy. J. Hepatol..

[98] Eun J.W., Yoon J.H., Ahn H.R., Kim S., Kim Y.B., Lim S.B., Park W., Kang T.W., Baek G.O., Yoon M.G. (2023). Cancer-associated fibroblast-derived secreted phosphoprotein 1 contributes to resistance of hepatocellular carcinoma to sorafenib and lenvatinib. Cancer Commun..

[99] Wang X., Yuan Z., Li Z., He X., Zhang Y., Wang X., Su J., Wu X., Li M., Du F. (2024). Key oncogenic signaling pathways affecting tumor-infiltrating lymphocytes infiltration in hepatocellular carcinoma: Basic principles and recent advances. Front. Immunol..

[100] Gao F., Xie K., Xiang Q., Qin Y., Chen P., Wan H., Deng Y., Huang J., Wu H. (2021). The density of tumor-infiltrating lymphocytes and prognosis in resectable hepatocellular carcinoma: A two-phase study. Aging.

[101] Stulpinas R., Zilenaite-Petrulaitiene D., Rasmusson A., Gulla A., Grigonyte A., Strupas K., Laurinavicius A. (2023). Prognostic Value of CD8+ Lymphocytes in Hepatocellular Carcinoma and Perineoplastic Parenchyma Assessed by Interface Density Profiles in Liver Resection Samples. Cancers.

[102] Chen L., Huang H., Huang Z., Chen J., Liu Y., Wu Y., Li A., Ge J., Fang Z., Xu B. (2023). Prognostic values of tissue-resident CD8^+^T cells in human hepatocellular carcinoma and intrahepatic cholangiocarcinoma. World J. Surg. Oncol..

[103] Sangro B., Sarobe P., Hervás-Stubbs S., Melero I. (2021). Advances in immunotherapy for hepatocellular carcinoma. Nat. Rev. Gastroenterol. Hepatol..

[104] Li S., Li K., Wang K., Yu H., Wang X., Shi M., Liang Z., Yang Z., Hu Y., Li Y. (2023). Low-dose radiotherapy combined with dual PD-L1 and VEGFA blockade elicits antitumor response in hepatocellular carcinoma mediated by activated intratumoral CD8^+^exhausted-like T cells. Nat. Commun..

[105] Wherry E.J., Ha S.J., Kaech S.M., Haining W.N., Sarkar S., Kalia V., Subramaniam S., Blattman J.N., Barber D.L., Ahmed R. (2007). Molecular signature of CD8+ T cell exhaustion during chronic viral infection. Immunity.

[106] Bai Y., Chen D., Cheng C., Li Z., Chi H., Zhang Y., Zhang X., Tang S., Zhao Q., Ang B. (2022). Immunosuppressive landscape in hepatocellular carcinoma revealed by single-cell sequencing. Front. Immunol..

[107] Gu J., Zhou J., Chen Q., Xu X., Gao J., Li X., Shao Q., Zhou B., Zhou H., Wei S. (2022). Tumor metabolite lactate promotes tumorigenesis by modulating MOESIN lactylation and enhancing TGF-β signaling in regulatory Tcells. Cell Rep..

[108] Kamm D.R., McCommis K.S. (2022). Hepatic stellate cells in physiology and pathology. J. Physiol..

[109] Delgado M.E., Cárdenas B.I., Farran N., Fernandez M. (2021). Metabolic reprogramming of liver fibrosis. Cells.

[110] Cassim Bawa F.N., Zhang Y. (2023). Retinoic acid signaling in fatty liver disease. Liver Res..

[111] Friedman S.L. (2008). Hepatic stellate cells: Protean, multifunctional, and enigmatic cells of the liver. Physiol. Rev..

[112] Pei Q., Yi Q., Tang L. (2023). Liver Fibrosis Resolution: From Molecular Mechanisms to Therapeutic Opportunities. Int. J. Mol. Sci..

[113] Chang M.L., Yang S.S. (2019). Metabolic signature of hepatic fibrosis: From individual pathways to systems biology. Cells.

[114] Otto J., Verwaayen A., Penners C., Hundertmark J., Lin C., Kallen C., Paffen D., Otto T., Berger H., Tacke F. (2023). Expression of Cyclin E1 in hepatic stellate cells is critical for the induction and progression of liver fibrosis and hepatocellular carcinoma in mice. Cell Death Dis..

[115] Ali E., Trailin A., Ambrozkiewicz F., Liška V., Hemminki K. (2022). Activated Hepatic Stellate Cells in Hepatocellular Carcinoma: Their Role as a Potential Target for Future Therapies. Int. J. Mol. Sci..

[116] Myojin Y., Hikita H., Sugiyama M., Sasaki Y., Fukumoto K., Sakane S., Makino Y., Takemura N., Yamada R., Shigekawa M. (2021). Hepatic Stellate Cells in Hepatocellular Carcinoma Promote Tumor Growth Via Growth Differentiation Factor 15 Production. Gastroenterology.

[117] Cogliati B., Yashaswini C.N., Wang S., Sia D., Friedman S.L. (2023). Friend or foe? The elusive role of hepatic stellate cells in liver cancer. Nat. Rev. Gastroenterol. Hepatol..

[118] Quiroz Reyes A.G., Lozano Sepulveda S.A., Martinez-Acuña N., Islas J.F., Gonzalez P.D., Heredia Torres T.G., Perez J.R., Garza Treviño E.N. (2023). Cancer Stem Cell and Hepatic Stellate Cells in Hepatocellular Carcinoma. Technol. Cancer Res. Treat..

[119] Wang Y., Zhang T., Sun M., Ji X., Xie M., Huang W., XIA L. (2021). Therapeutic values of myeloid-derived suppressor cells in hepatocellular carcinoma: Facts and hopes. Cancers.

[120] Lu L.C., Chang C.J., Hsu C.H. (2019). Targeting myeloid-derived suppressor cells in the treatment of hepatocellular carcinoma: Current state and future perspectives. J. Hepatocell. Carcinoma.

[121] Weston C.J., Zimmermann H.W., Adams D.H. (2019). The role of myeloid-derived cells in the progression of liver disease. Front. Immunol..

[122] Li T., Zhang X., Lv Z., Gao L., Yan H. (2020). Increased Expression of Myeloid-Derived Suppressor Cells in Patients with HBV-Related Hepatocellular Carcinoma. Biomed. Res. Int..

[123] Zhang X., Fu X., Li T., Yan H. (2019). The prognostic value of myeloid derived suppressor cell level in hepatocellular carcinoma: A systematic review and meta-analysis. PLoS ONE.

[124] Zhou S.L., Yin D., Hu Z.Q., Luo C.B., Zhou Z.J., Xin H.Y., Yang X.R., Shi Y.H., Wang Z., Huang X.W. (2019). A Positive Feedback Loop Between Cancer Stem-Like Cells and Tumor-Associated Neutrophils Controls Hepatocellular Carcinoma Progression. Hepatology.

[125] Arvanitakis K., Mitroulis I., Germanidis G. (2021). Tumor-associated neutrophils in hepatocellular carcinoma pathogenesis, prognosis, and therapy. Cancers.

[126] Ramon-Gil E., Geh D., Leslie J. (2023). Harnessing neutrophil plasticity for HCC immunotherapy. Essays Biochem..

[127] Pan J.J., Xie S.Z., Zheng X., Xu J.F., Xu H., Yin R.Q., Luo Y.L., Shen L., Chen Z.R., Chen Y.R. (2024). Acetyl-CoA metabolic accumulation promotes hepatocellular carcinoma metastasis via enhancing CXCL1-dependent infiltration of tumor-associated neutrophils. Cancer Lett..

[128] Galdiero M.R., Bonavita E., Barajon I., Garlanda C., Mantovani A., Jaillon S. (2013). Tumor associated macrophages and neutrophils in cancer. Immunobiology.

[129] Leslie J., Mackey J.B.G., Jamieson T., Ramon-Gil E., Drake T.M., Fercoq F., Clark W., Gilroy K., Hedley A., Nixon C. (2022). CXCR2 inhibition enables NASH-HCC immunotherapy. Gut.

[130] Kong J., Kong L., Kong J., Ke S., Gao J., Ding X., Zheng L., Sun H., Sun W. (2012). After insufficient radiofrequency ablation, tumor-associated endothelial cells exhibit enhanced angiogenesis and promote invasiveness of residual hepatocellular carcinoma. J. Transl. Med..

[131] Ye B.G., Sun H.C., Zhu X.D., Chai Z.T., Zhang Y.Y., Ao J.Y., Cai H., Ma D.-N., Wang C.-H., Qin C.-D. (2016). Reduced expression of CD109 in tumor-associated endothelial cells promotes tumor progression by paracrine interleukin-8 in hepatocellular carcinoma. Oncotarget.

[132] Crusio W.E., Lambris J.D., Radeke H.H. Advances in Experimental Medicine and Biology. Volume 1234 Series Editors [Internet]. http://www.springer.com/series/5584.

[133] Leone P., Malerba E., Susca N., Favoino E., Perosa F., Brunori G., Prete M., Racanelli V. (2024). Endothelial cells in tumor microenvironment: Insights and perspectives. Front. Immunol..

[134] Yao X., Zeng Y. (2023). Tumour associated endothelial cells: Origin, characteristics and role in metastasis and anti-angiogenic resistance. Front. Physiol..

[135] Kaps L., Schuppan D. (2020). Targeting cancer associated fibroblasts in liver fibrosis and liver cancer using nanocarriers. Cells.

[136] Jeng K.S., Sheen I.S., Lin S.S., Leu C.M., Chang C.F. (2021). The role of endoglin in hepatocellular carcinoma. Int. J. Mol. Sci..

[137] Zhang L., Zhang Q., Teng D., Guo M., Tang K., Wang Z., Wei X., Lin L., Zhang X., Wang X. (2023). FGF9 Recruits β-Catenin to Increase Hepatic ECMSynthesis Promote NASH-Driven HCC. Adv. Sci..

[138] Roy A.M., Iyer R., Chakraborty S. (2023). The extracellular matrix in hepatocellular carcinoma: Mechanisms and therapeutic vulnerability. Cell Rep. Med..

[139] Okabe H., Hayashi H., Nakagawa S., Imai K., Nitta H., Arima K., Hashimoto D., Chikamoto A., Ishiko T., Beppu T. (2016). Inducible factors for cancer-associated fibroblasts in liver cancer versus myofibroblasts in inflammatory liver disease. Histol Histopathol..

[140] Filliol A., Schwabe R.F. (2019). Contributions of Fibroblasts, Extracellular Matrix, Stiffness, and Mechanosensing to Hepatocarcinogenesis. Semin. Liver Dis..

[141] Pham L., Kyritsi K., Zhou T., Ceci L., Baiocchi L., Kennedy L., Chakraborty S., Glaser S., Francis H., Alpini G. (2022). The Functional Roles of Immune Cells in Primary Liver Cancer. Am. J. Pathol..

[142] Sachdeva M., Arora S.K. (2020). Prognostic role of immune cells in hepatocellular carcinoma. EXCLI J..

[143] Fasano R., Shadbad M.A., Brunetti O., Argentiero A., Calabrese A., Nardulli P., Calbi R., Baradaran B., Silvestris N. (2021). Immunotherapy for Hepatocellular Carcinoma: New Prospects for the Cancer Therapy. Life.

[144] Teng C.F., Wang T., Wu T.H., Lin J.H., Shih F.Y., Shyu W.C., Jeng L.B. (2020). Combination therapy with dendritic cell vaccine and programmed death ligand 1 immune checkpoint inhibitor for hepatocellular carcinoma in an orthotopic mouse model. Ther. Adv. Med. Oncol..

[145] Chen C., Wang Z., Ding Y., Qin Y. (2023). Tumor microenvironment-mediated immune evasion in hepatocellular carcinoma. Front. Immunol..

[146] Wang C.I., Chu P.M., Chen Y.L., Lin Y.H., Chen C.Y. (2021). Chemotherapeutic drug-regulated cytokines might influence therapeutic efficacy in hcc. Int. J. Mol. Sci..

[147] Lin Y.D., Wu G.S., Rao M.Y., Liu Y.H., Han Y.W., Zhang J., Zhang J.W. (2024). Effect of cytokines on advanced hepatocellular carcinoma prognosis receiving radiotherapy and tislelizumab plus anlotinib: A single-center phase II clinical trial. Sci. Rep..

[148] Dewidar B., Meyer C., Dooley S., Meindl-Beinker N. (2019). Tgf-β in hepatic stellate cell activation and liver fibrogenesis—Updated 2019. Cells.

[149] García-Pras E., Fernández-Iglesias A., Gracia-Sancho J., Pérez-Del-pulgar S. (2022). Cell death in hepatocellular carcinoma: Pathogenesis and therapeutic opportunities. Cancers.

[150] Pocino K., Stefanile A., Basile V., Napodano C., D’Ambrosio F., Di Santo R., Callà C.A.M., Gulli F., Saporito R., Ciasca G. (2023). Cytokines and Hepatocellular Carcinoma: Biomarkers of a Deadly Embrace. J. Pers. Med..

[151] Alqahtani A., Khan Z., Alloghbi A., Ahmed T.S.S., Ashraf M., Hammouda D.M. (2019). Hepatocellular carcinoma: Molecular mechanisms and targeted therapies. Medicina.

[152] Jing Y., Sun K., Liu W., Sheng D., Zhao S., Gao L., Wei L. (2018). Tumor necrosis factor-α promotes hepatocellular carcinogenesis through the activation of hepatic progenitor cells. Cancer Lett..

[153] Kumar V., Kaur J., Anand Pawar V., Wang Y., Zhang Y. (2022). IL-32 and IL-34 in hepatocellular carcinoma. Front. Med..

[154] Sakamoto Y., Yoshio S., Doi H., Mori T., Matsuda M., Kawai H., Shimagaki T., Yoshikawa S., Aoki Y., Osawa Y. (2021). Increased Frequency of Dysfunctional Siglec-7−CD57+PD-1+ Natural Killer Cells in Patients With Non-alcoholic Fatty Liver Disease. Front. Immunol..

[155] Gao Y., Guo J., Bao X., Xiong F., Ma Y., Tan B., Yu L., Zhao Y., Lu J. (2021). Adoptive Transfer of Autologous Invariant Natural Killer T Cells as Immunotherapy for Advanced Hepatocellular Carcinoma: A Phase I Clinical Trial. Oncologist.

[156] Pan X., Kaminga A.C., Wen S.W., Liu A. (2020). Chemokines in hepatocellular carcinoma: A meta-analysis. Carcinogenesis.

[157] Wang Y., Huang J., Tian Z., Zhou Y., Yang J. (2020). The role of CXC cytokines as biomarkers and potential targets in hepatocellular carcinoma. Math. Biosci. Eng..

